# Abnormal alterations of regional spontaneous neuronal activity and functional connectivity in insomnia patients with difficulty falling asleep: a resting-state fMRI study

**DOI:** 10.1186/s12883-023-03481-3

**Published:** 2023-12-04

**Authors:** Tongfei Jiang, Xuejiao Yin, Liying Zhu, Weilin Jia, Zhongjian Tan, Bin Li, Jing Guo

**Affiliations:** 1 Department of Acupuncture and Moxibustion, Beijing Hospital of Traditional Chinese Medicine, Capital Medical University, Beijing Key Laboratory of Acupuncture Neuromodulation, Beijing, 100010 China; 2 Graduate School, Beijing University of Chinese Medicine, Beijing, 100029 China; 3 Department of Radiology, Dong Zhimen Hospital Beijing University of Chinese Medicine, Beijing, 100010 China

**Keywords:** Insomnia disorder, Sleep latency (SL), Hyperarousal, Functional magnetic resonance imaging (fMRI), Amplitude of low-frequency fluctuation (ALFF), Functional connectivity (FC)

## Abstract

**Background:**

Insomnia disorder (ID) seriously affects people’s daily life. Difficulty falling asleep is the most commonly reported complaint in patients with ID. However, the mechanism of prolonged sleep latency (SL) is still obscure. The aim of our present study was to investigate the relationship between prolonged SL and alterations in spontaneous neural activity and brain functional connectivity (FC) in ID patients using functional magnetic resonance imaging (fMRI).

**Methods:**

A total of 52 insomniacs with difficulty falling asleep and 30 matched healthy controls (HCs) underwent resting-state fMRI. The amplitude of low-frequency fluctuation (ALFF) was measured and group differences were compared. The peak areas with significantly different ALFF values were identified as the seed regions to calculate FC to the whole brain. SL was assessed by a wrist actigraphy device in ID patients. The Pittsburgh Sleep Quality Index (PSQI), Hamilton Anxiety Rating Scale (HAMA), and Hyperarousal Scale (HAS) were evaluated in both ID patients and HCs. Finally, correlation analyses were performed between the clinical features and FC/ALFF values.

**Results:**

ID patients showed higher PSQI, HAMA, HAS scores than HCs. The functional MRI results indicated increased ALFF value in the left insula and right amygdala and decreased ALFF value in the right superior parietal lobe (SPL) in ID patients. The seed-based FC analysis demonstrated increased FC between the left insula and the bilateral precentral gyrus and FC between the right amygdala and the left posterior cingulate cortex (PCC) in patients with ID. Correlation analysis indicated that the increased FC value of the right amygdala-left PCC was positively correlated with SL measured by actigraphy.

**Conclusion:**

This study revealed abnormal regional spontaneous fluctuations in the right amygdala, left insula, and right SPL, as well as increased FC in the left insula-precentral and right amygdala-left PCC. Moreover, the prolonged SL was positively correlated with the abnormal FC in the right amygdala-left PCC in ID patients. The current study showed the correlation between prolonged SL and the abnormal function of emotion-related brain regions in ID patients, which may contribute to a better understanding of the neural mechanisms underlying difficulty falling asleep in patients with ID.

**Clinical Trial Registration:**

http://www.chictr.org.cn., ChiCTR1800015282. Registered on 20th March 2018.

**Supplementary Information:**

The online version contains supplementary material available at 10.1186/s12883-023-03481-3.

## Introduction

Insomnia disorder (ID) is mainly marked by difficulty in falling asleep, maintaining sleep, and early morning awakening, coupled with significantly impaired daytime functioning [1]. ID has affected approximately 22.1% of the global population, and the prevalence of difficulty falling asleep has already reached 13.4% worldwide [2, 3]. Moreover, it was shown that people with sleep initiation problems reported the worst sleep quality and more severe daytime dysfunction than other insomnia subtypes [4]. Prolonged sleep latency (SL) may promote poor sleep habits such as watching TV or phones in bed, which in turn makes it more difficult to fall asleep. Difficulty with sleep onset will reduce sleep time, disrupt circadian rhythms, and reduce work efficiency [5, 6]. Prolonged SL has been found to be related to vegetative function disturbances, e.g., decreased heart rate variability and increased body temperature [7, 8]. In addition, there are indications that prolonged SL plays an important role in affective disturbances and even psychological disorders [9]. Thus, it is necessary to explore the mechanism that inhibits sleep onset in patients with ID.

It is indicated that difficulty falling asleep occurs as a result of increased cognitive arousal, which mainly refers to heightened vigilance against external threats and excessive anxiety from inner impulses [10–12]. ID patients usually experience 24-hour cognitive hyperarousal, which is mainly reflected in abnormal mental traits such as anxiety, depression, and vigilance [13].Abnormal cognitive and vigilance states will induce bedtime worry and rumination, which are key psychiatric factors responsible for the inability to fall asleep [4]. Studies also found that spontaneous, intrusive thoughts or anxious minds would further prolong SL time [14, 15]. Healthy sleep is a spontaneous process with automatic and unconscious control; however, when ID patients focus their attention on sleep initiation and make efforts to fall asleep, they are in a state of cognitive hyperarousal that makes falling asleep more difficult [16]. Although abnormal psychophysiological features have been found to partly explain the difficulty falling asleep in ID patients, the central neuroimaging changes related to prolonged SL are worth further exploring.

The resting-state functional magnetic resonance imaging (rs-fMRI) technique provides a useful avenue to detect neurobiological alterations, which depict changes in blood oxygen level dependent (BOLD) consequent to spontaneous modulation of neural metabolism [17]. Neuroimaging studies have revealed that insomnia patients presented a significantly higher ALFF value in the posterior cingulate cortex (PCC), hippocampus, putamen, and anterior insula cortex, whereas a significantly lower ALFF value was found in the superior parietal lobe (SPL) and inferior frontal gyrus [18–20]. It has also been proven that mind wandering and visual thoughts during sleep onset are strongly related to the default mode network (DMN) and visual network [21, 22]. Yang et al. found that the functional connectivity (FC) between the DMN and visual network was weakened while the information exchange within the visual network was enhanced in people who suffered from sleep initiation problems [23]. Previous neuroimaging studies have reported that abnormal functional activity in patients with ID is mainly in brain regions related to emotion awareness, cognition processes, and goal-directed behavior, including the frontal gyrus, amygdala, cingulate cortex, insula, and hippocampus [20, 24, 25]. However, fMRI studies focusing on the neuroimaging mechanisms of SL in ID patients who have difficulties with sleep initiation are still limited.

The amplitude of low-frequency fluctuation (ALFF), fractional amplitude of low-frequency fluctuation (fALFF) are frequently used measures in fMRI studies to reflect the local BOLD signal. ALFF measures the total power of a given time course within a specific frequency range (e.g., 0.01–0.10 Hz), which is considered an effective approach to detect the regional intensity of spontaneous fluctuations in the BOLD signal of brain regions [26, 27]. As a normalized index of ALFF, fALFF can improve the sensitivity for the detection of spontaneous brain activities by surpassing the physiological noise [28]. However, as a proportional measure, fALFF had generally lower test-retest reliability than ALFF, so to some extent, the reliability of ALFF is higher than that of fALFF [26, 29]. Moreover, prior studies had consistently demonstrated that the ALFF was more detectable within gray matter than fALFF [28, 30]. Functional connectivity (FC) analysis represents the temporal correlation between discrete or continuous time series of brain regions, and it may identify brain regions that are synchronized in activity [31–33]. Seed-based FC could investigate more details of the connectivity patterns, which may lead to a better understanding of the difficulty of falling asleep [34].

As a wearable sleep device for sleep quality monitoring, actigraphy is a simple and convenient objective indicator that reflects latency to sleep, wake time after sleep onset, and total sleep time of the wearer [35]. The actigraphy unit can infer sleep from the presence or absence of wrist movements, and its accuracy has been well validated using polysomnography [36].

Using a multiple-algorithm analysis in combination of ALFF and FC, we conducted a data-driven study to explore the spontaneous brain activity and seed-based FC in ID patients with difficulty falling asleep. According to previous neuroimaging studies, correlation analysis between brain activation changes and clinical data is one of the most commonly used methods to reveal the neuroimaging mechanisms of disorders such as insomnia [37], depression [38], and generalized anxiety disorder [39]. In the current study, we collected SL time in ID patients using wrist actigraphy devices and further explored the relationship between prolonged SL and alterations in ALFF value and seed-based FC value. This study may shed light on the potential neuroimaging mechanisms underlying difficulty falling asleep in ID.

## Materials and methods

### Participants

**Fig. 1:**
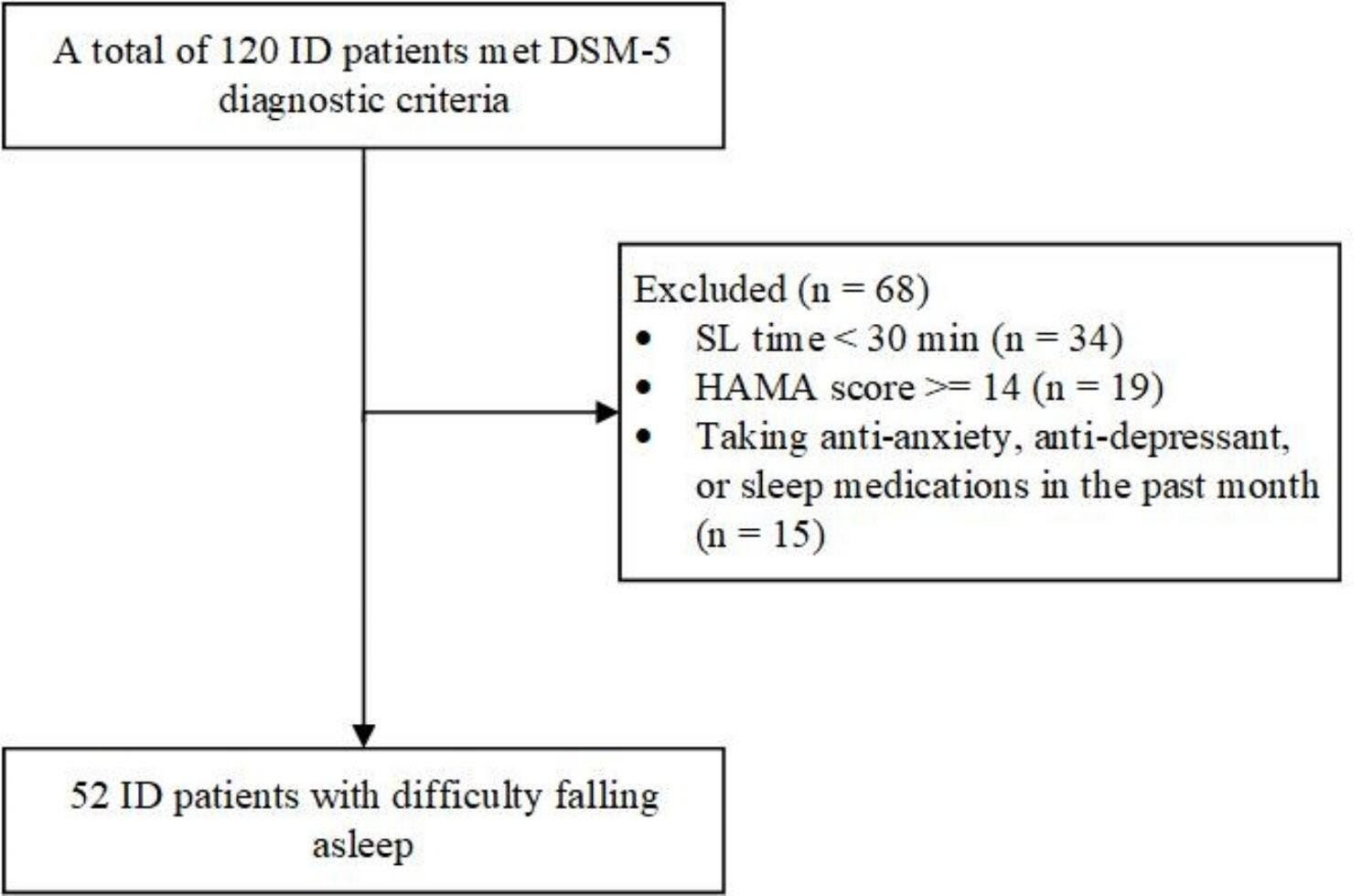
Flowchart for inclusion of ID patients with difficulty falling asleep

Fifty-two ID patients who had difficulty falling asleep were recruited from outpatient acupuncture clinics of Beijing Traditional Chinese Medicine (TCM) Hospital during the period from September 2019 to September 2021 (Fig. 1). ID patients were screened by an expert working at the neurology outpatient service of the Beijing TCM Hospital based on the fifth edition of the Diagnostic and Statistical Manual of Mental Disorders (DSM-5) [40]. In addition, we also recruited 30 age-, education-, and sex-matched healthy participants from the local community to serve as the control group through advertising posters. Our inclusion criteria for ID patients with difficulty falling asleep were as follows: (i) age 18–60 years; (ii) PSQI score [41] > 8; (iii) SL time > = 30 min [40]; (iv) Hamilton Anxiety Rating Scale (HAMA) score [42] < 14; (v) informed consent signed by each participant; (vi) not taking anti-anxiety, anti-depressant, or sleep medications in the past month; and (vii) right-handedness. ID patients were excluded from the study if any of the following criteria were met: (i) depression, anxiety, schizophrenia, or other serious mental illnesses; (ii) severe heart, brain, kidney, or liver disease; (iii) failure to cooperate with examination; (iv) apnea syndrome; (v) pregnant and lactating women; (vi) claustrophobia or other contraindications to MRI examination; or (vii) a definite lesion on MRI or grossly asymmetrical anatomy of the head. Healthy controls (HCs) were included in the study according to the following criteria: (i) they had good sleep habits and PSQI score < 3; (ii) HAMA score < 7; (iii) Hyperarousal Scale (HAS) [43] score < 32; (iv) no serious heart, lung, kidney, or neuropsychological disease; (v) no structural abnormalities on conventional brain magnetic resonance imaging (MRI); (vi) females were not pregnant or nursing; and (vii) right-handedness.

### Methods

#### Demographics and clinical characteristics

Demographics, including age, sex, and education level, of ID patients and HCs were collected. In this study, clinical characteristics were focused on sleep quality, arousal, and anxiety symptoms. All participants were required to complete tests, including: (1) the PSQI scale to evaluate sleep quality, with higher scores indicating worse sleep quality [44]; (2) the HAS to reflect cortical arousal level, with higher scores indicating a higher level of cortical arousal [43]; and (3) the HAMA scale to assess the severity of anxiety [45].

In order to detect SL, ID patients were requested to wear an actigraphy unit (MTI Health Services Company, Pensacola, FL, USA) on their left wrist before going to bed and to take it off in the morning after waking up. In the current study, ID patients were required to wear the actigraphy device to sleep for 7 consecutive nights, and they were not allowed to do anything unrelated to falling asleep while wearing the actigraphy unit. After data collection was completed, the 7-night SL of each ID patient was extracted, and the average was taken as the mean SL time of each ID patient.

#### MRI data acquisition

The resting-state MRI/fMRI scans were acquired using 3.0 T MRI scanner (Siemens Magnetom Verio, Erlangen, Germany) with a 24-channel phased-array head coil at Dong Zhimen Hospital affiliated to Beijing University of Chinese Medicine. All MRI/fMRI scans were conducted between 9:00 am and 11:30 am by the same skilled and professional technician. All participants underwent MRI/fMRI scans on the day of clinical characteristic assessment. Each participant was supine and their head was held tightly in place with foam pads. All participants were required to relax, remain motionless, and keep their eyes open to avoid falling asleep. The scanner could observe the participant’s face through the monitor, and if their eyes were closed, the participant would be reminded through the microphone to open their eyes and stay awake. High-resolution brain T1-weighted brain anatomical images were collected using a volumetric three-dimensional magnetization prepared by a rapid-acquisition gradient-echo sequence with the following parameters: repetition time (TR) = 2000 ms, echo time (TE) = 3.51 ms, flip angle (FA) = 7°, slice number = 188, slice thickness/gap = 1.0/0 mm, voxel size = 1.0 mm × 1.0 mm × 1.0 mm, matrix = 256 × 256, and field of view (FOV) = 256 mm × 256 mm. Functional data were collected by an echo planar imaging (EPI) sequence with scan parameters of TR = 2000 ms, TE = 30 ms, FA = 90°, slice number = 32, slice thickness/gap = 3.5/0.6 mm, voxel size = 3.5 mm × 3.5 mm × 3.5 mm, FOV = 224 mm × 224 mm, matrix size = 64 × 64, and phase-encoding direction: anterior > > posterior. In total, 240 volumes were acquired, and the EPI sequence scan time was 8 min.

#### Image data preprocessing

Image preprocessing and analysis were performed using DPABI V5.1_201201 (5.1, advanced edition, http://rfmri.org/dpabi) based on MATLAB_R2015b (Mathworks, Inc., Natick, MA) and SPM12 (Wellcome Trust Centre for Neuroimaging, London, UK, https://www.fil.ion.ucl.ac.uk/spm/) [46]. Briefly, the preprocessing consisted of the following steps: (1) removal of the first 10 volumes for signal equilibrium; (2) slice timing for acquisition delay; (3) correction of the head motion; participants were excluded if they had a maximum displacement of 3 mm in the x, y, or z axis and 3 degrees of angular motion [47]; (4) nuisance covariates regression to regress white matter, cerebrospinal fluid (CSF), and global signal; (5) normalization to Montreal Neurological Institute (MNI) space; (6) smoothing with [6, 6, 6] full width at half maximum; (7) removal of the linear trend to reduce artifacts related to the rising temperature of the MRI equipment; and (8) band-pass filtering (0.01–0.08 Hz) was performed to remove the influence of low-frequency drift and high-frequency noise [48]. In order to minimize the possible effect of head micromovements on ALFF and FC, framewise displacement (FD) values were calculated for each participant.

#### ALFF analysis

ALFF measures the ratio of the amplitude across the low-frequency band (0.01–0.08 Hz), which may effectively reflect intrinsic neuronal spontaneous activity [26, 28]. The time series of each voxel of the brain was transformed to a frequency domain with a fast Fourier transform [49]. The averaged square root of the amplitude within a specific low-frequency range (0.01–0.08 Hz) for each voxel was taken as the ALFF value [50]. In order to improve the normality, z-standardized ALFF (zALFF) maps were calculated for the following statistical analysis.

#### Seed-based FC analysis

To explore more details about resting-state FC alterations, a seed-based interregional correlation analysis was performed using the DPABI software package. The peak areas that had a significant difference in ALFF value between the two groups (ID patients’ ALFF vs. HCs’ ALFF) were identified as the seed regions, which were regions of interest (ROIs). Then, the Pearson correlation coefficient of the average time series between the ROI and every voxel of the whole brain was computed, and its value was the FC strength. Finally, Fisher’s z transformation (zFC) was used to improve the normality of correlation coefficients.

### Statistical analysis

#### Demographics and clinical data analysis

Demographic and clinical data statistics tests were performed using SPSS Statistics for Windows Version V21.0 (IBM SPSS Statistics, IBM Corp., Somers, New York, USA). The threshold for statistical significance was set at 0.05. The normality of the data was tested using the Shapiro-Wilk test. Continuous variables with normal distributions were analyzed using independent *t* test. For categorical variables, including sex ratios and education level, we used the chi-square (*x*^2^) test to compare the differences between ID patients and HCs. Moreover, Pearson’s correlation was used to assess concordance between objective SL (measured by actigraphy unit) and subjective SL (calculated by PSQI scale) [51].

#### ALFF and seed-based FC statistical analysis

Two-sample *t* test was performed to test the zALFF or zFC value difference between ID patients and HCs, with age, sex, education level, and FD value as covariates. The statistical map was corrected for multiple comparisons using a threshold set at *p* < 0.005 for voxel level and *p* < 0.05 for the cluster level, corrected with the false discovery rate (FDR).

#### Correlation analysis

To identify the association between the ALFF or FC and performance level on clinical tests, the mean ALFF and FC values for all voxels in significant areas were extracted separately. Pearson’s correlation analysis was performed using SPSS to investigate the relationship between the values of Fisher z ALFF or z FC and clinical data (including PSQI, HAMA, HAS, and SL), with age, sex, and education level as covariates. Bonferroni corrections were used for multiple comparisons. Both uncorrected and corrected *p* values were reported. The significance level was set at *p*_uncorrected_ < 0.0125 (0.05/4) and *p*_Bonferroni−corrected_ < 0.05 for the two-tailed test.

## Results

### Demographic and clinical characteristics

There were no significant between-group differences in demographic data, including age (*p* = 0.40), sex (*p* = 0.61), and education level (*p* = 0.81) between ID patients and HCs. As expected, ID patients had higher PSQI, HAMA, and HAS scores than HCs (Table 1). In addition, the results of the mean objective SL collected by the actigraphy unit and the subjective SL assessed by the PSQI are also shown in Table 1. Moreover, Pearson correlation results showed consistency between the SL measured by the actigraphy and the SL assessed by PSQI scale (r = 0.77, *p* < 0.001) (Table 2).

**Table 1:** Demographic and clinical characteristics of ID patients and HCs

Characteristic	ID patients (n = 52)	HC (n = 30)	*t* value	*p* value
Age (year) ^a^	36.81 ± 9.60	35.23 ± 7.46	0.81	0.40
Sex ^b^				0.61
Male	17	7		
Female	35	23		
Education level ^b^				0.81
Above Bachelor’s degree	34	19		
Below Bachelor’s degree	18	11		
PSQI ^a^	12.19 ± 2.30	1.00 ± 0.87	31.19	< 0.01^*^
SL (PSQI, min)	80.43 ± 38.04	14.30 ± 6.33	11.55	< 0.01^*^
HAMA ^a^	10.79 ± 2.42	1.23 ± 1.00	24.68	< 0.01^*^
HAS ^a^	43.60 ± 7.61	7.47 ± 3.80	28.93	< 0.01^*^
SL (actigraphy, min)	73.03 ± 39.16	-	-	-

*Notes*: Except for sex and education level, data for other characteristics are described as mean ± standard deviation. Abbreviations: PSQI, Pittsburgh Sleep Quality Index; HAMA, Hamilton Anxiety Rating Scale; HAS, Hyperarousal Scale; SL, sleep latency

^*^Significant difference between two groups

^a^Two-sample t test

^b^Chi-squared test

**Table 2:** Correlation analysis between SL measured by actigraphy and SL assessed by PSQI scale

	SL (PSQI, min)	SL (actigraphy, min)	r	*p*
ID patients	80.43 ± 38.04	73.03 ± 39.16	0.77	< 0.001

Abbreviations: SL, sleep latency; PSQI, Pittsburgh Sleep Quality Index

### Alterations of ALFF in ID patients

**Fig. 2:**
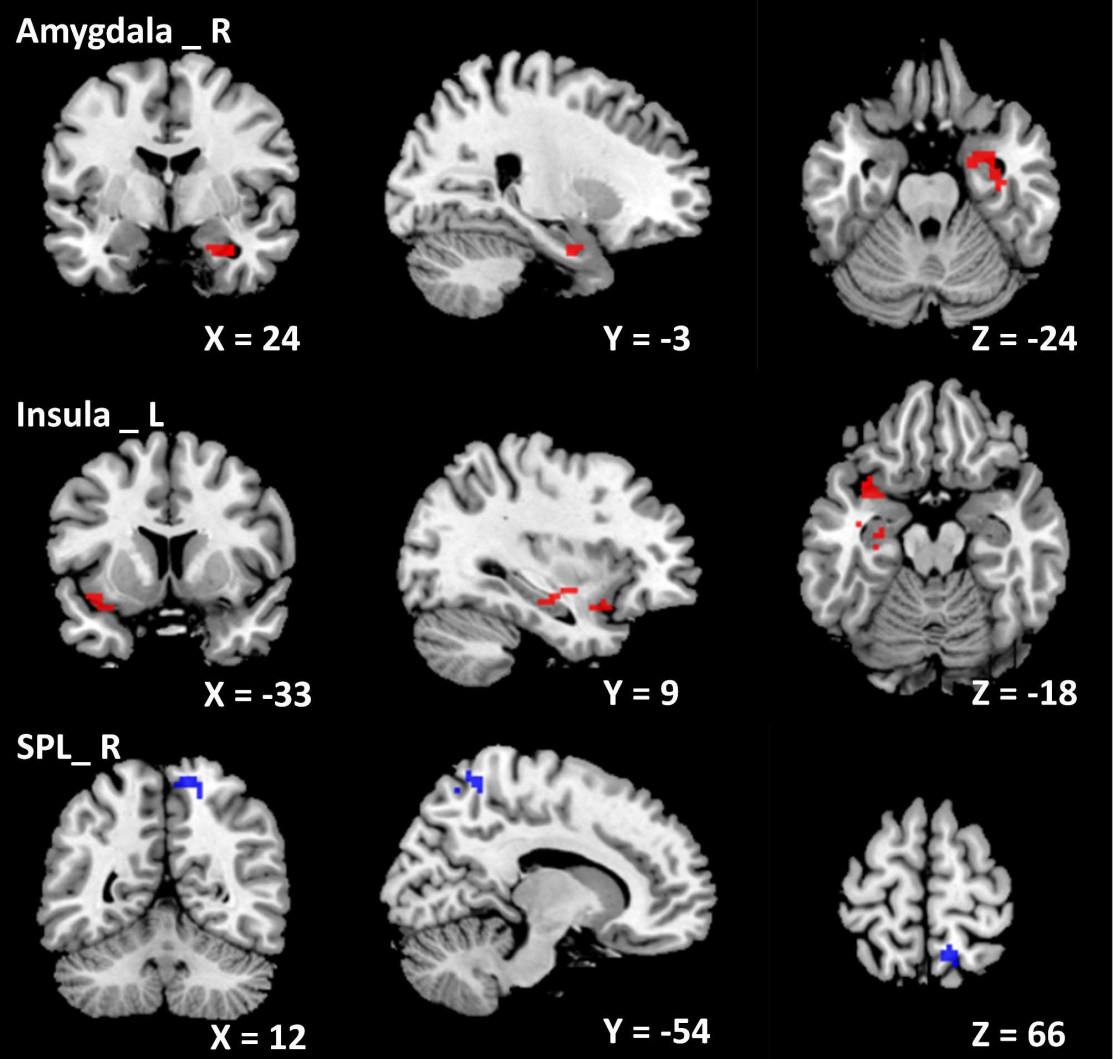
Cortical areas of increased/decreased ALFF in ID patients compared with HC. The red color shows increased spontaneous functional activity in the right amygdala and the left insula in ID patients. The bule color shows decreased spontaneous functional activity in the right superior parietal lobule in insomnia patients. SPL, superior parietal lobule; L, left; R, right

Compared with HCs, ID patients had significantly increased ALFF in the left insula (*p* = 0.036) and right amygdala (*p* = 0.039), as well as decreased ALFF in the right SPL (*p* = 0.005) at the threshold we set (cluster-level *p* < 0.05, FDR corrected) (Table 3; Fig. 2).

**Table 3:** Brain regions with abnormal ALFF value in ID patients compared with HCs

Comparison	Brain area	Cluster size	MNI coordinates			*t* value	*p* value
			x	y	z		
ID patients > HC	Insula _ L	89	-33	9	-18	3.80	0.036
	Amygdala _ R	70	24	-3	-24	3.47	0.039
ID patients < HC	SPL _ R	126	12	-54	66	3.64	0.005

*Notes*: Using two-sample *t* test, *p <* 0.05 (cluster-level FDR corrected) represents statistical significance. ID, insomnia disorder; HCs, healthy controls; SPL, superior parietal lobule; L, left; R, right

### Alterations of seed-based FC in ID patients

**Fig. 3:**
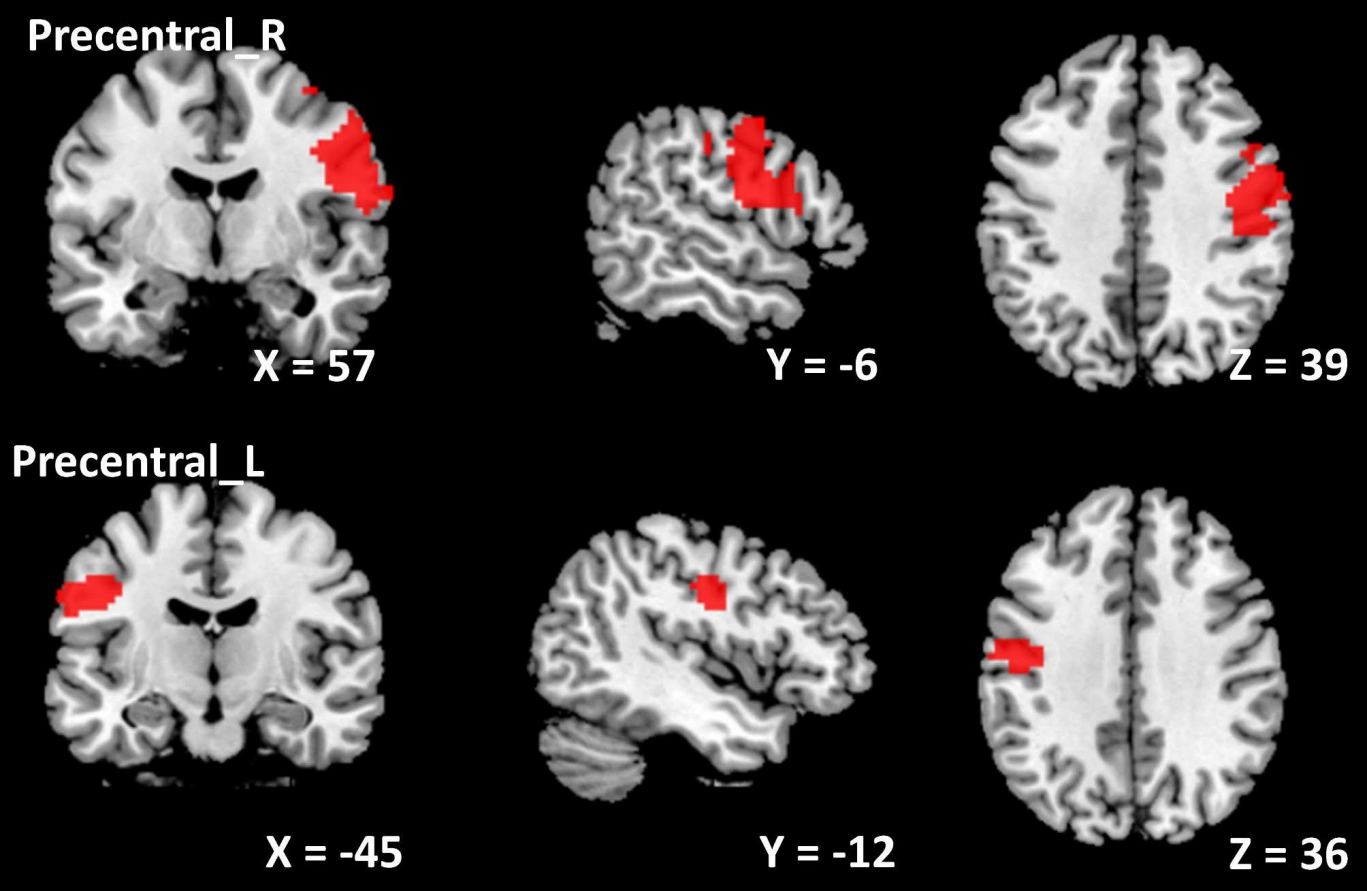
The ID patients showed increased connectivity between the left insula and bilateral precentral gyrus (right precentral: x = 57, y = -6, z = 39; left precentral: x = -45, y = -12, z = 36). L, left; R, right

**Fig. 4:**
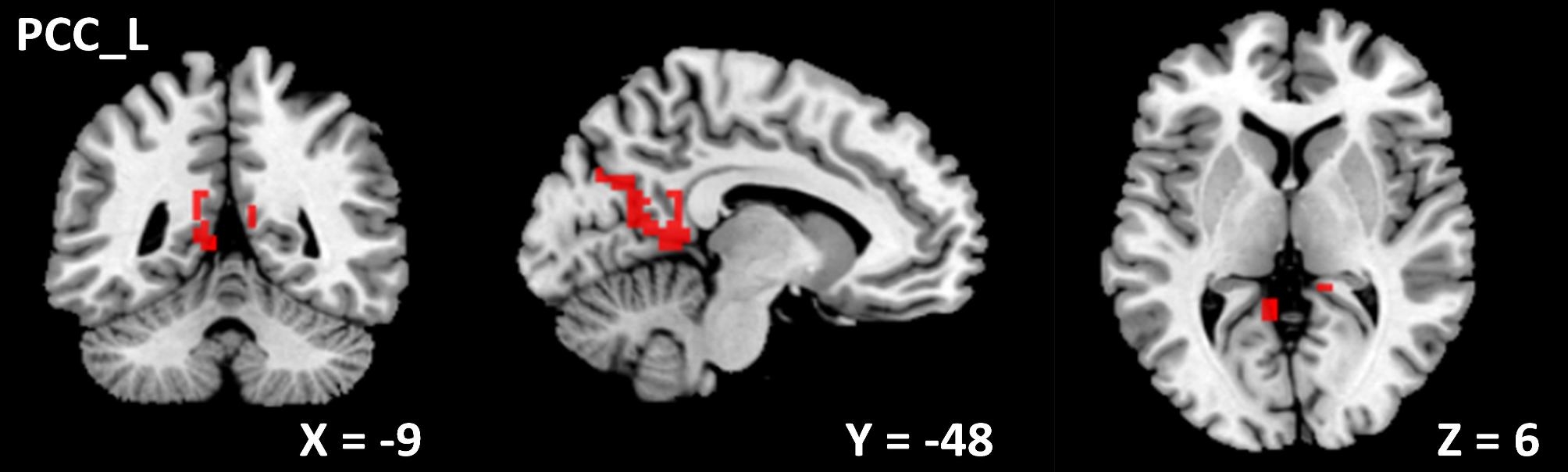
The ID patients showed increased connectivity between the right amygdala and left PCC (x = -9, y = -48, z = 6). PCC, posterior cingulate cortex; L, left

The center points of the peak *t* value in brain regions that showed significant differences in ALFF between ID patients and HCs (left insula, right amygdala, and right SPL) were defined as spherical ROIs (r = 3 mm). The FC analysis revealed increased connectivity between the left insula and bilateral precentral (Table 4; Fig. 3) and increased connectivity between the right amygdala and left PCC in the ID patients (FDR correction, *p* < 0.05) (Table 4; Fig. 4). However, ID patients failed to reveal any suprathreshold clusters between the ROI of the right SPL and the whole brain regions.

**Table 4:** Abnormal FC in ID patients compared with HCs

Comparison	ROI	Connected region	Cluster size	MNI coordinates			*t* value	*p* value
				x	y	z		
ID patients > HC	Insula _ L	Precentral _ R	886	57	-6	39	4.94	< 0.001
		Precentral _ L	164	-45	-12	36	4.18	< 0.001
	Amygdala _ R	PCC _ L	298	-9	-48	6	3.46	0.004

*Notes*: Using two-sample *t* test, *p <* 0.05 (cluster level FDR corrected) represents statistical significance. ROI, region of interest; PCC, posterior cingulate cortex; HCs, healthy controls; L, left; R, right

### Correlation results

**Fig. 5:**
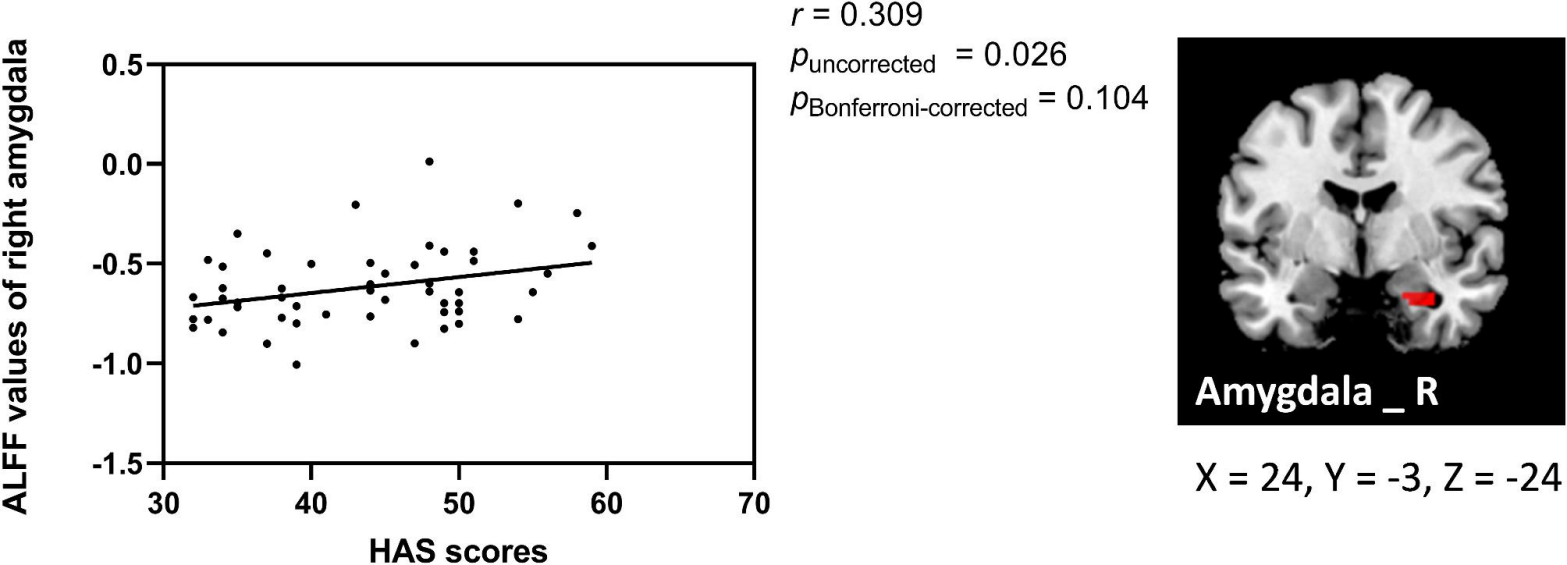
There was a positive trend in ID patients between increased ALFF value in the right amygdala and HAS scores (*r* = 0.309, *p*_uncorrected_ = 0.026, *p*_Bonferroni−corrected_ = 0.104)

**Fig. 6:**
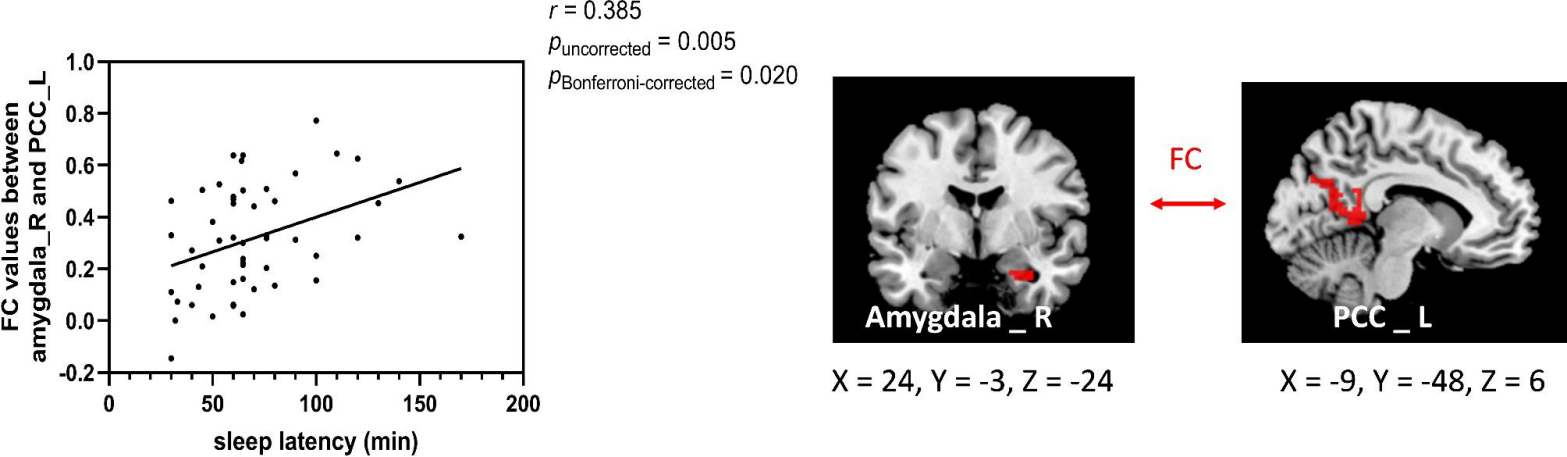
The significant positive correlation in ID patients between increased FC values and SL measured by actigraphy (the right amygdala and left PCC, *r* = 0.385, *p*_uncorrected_ = 0.005, *p*_Bonferroni−corrected_ = 0.020)

Correlation analysis was conducted with age, sex, and education level as covariates. We found that the increased ALFF value in the right amygdala was positively correlated with the HAS score (*r* = 0.309, *p*_uncorrected_ = 0.026, *p*_Bonferroni−corrected_ = 0.104) (Fig. 5), and the increased FC between the right amygdala and left PCC was positively correlated with SL measured by actigraphy (*r* = 0.385, *p*_uncorrected_ = 0.005, *p*_Bonferroni−corrected_ = 0.020) (Fig. 6). The correlation between the increased ALFF value of the right amygdala and the HAS scores did not survive after Bonferroni correction but showed a significant trend. No correlation was found between other FC/ALFF value and clinical data (shown in Supplementary Tables S1 and S2, all *p*_uncorrected_ > 0.05).

Additional analysis was performed to determine whether the correlation between the functional alterations and clinical data was significant when HAMA was included as one of the covariates. Results showed that the positive correlation between increased FC in the right amygdala-left PCC and SL measured by actigraphy still survived after the addition of the HAMA score as a covariate (*r* = 0.403, *p*_uncorrected_ = 0.003, *p*_Bonferroni−corrected_ = 0.009). And after adding the HAMA score as a covariate, the positive correlation trend between the increased ALFF value in the right amygdala and the HAS score was consistent with the preceding results (*r* = 0.306, *p*_uncorrected_ = 0.020, *p*_Bonferroni−corrected_ = 0.060) (shown in Supplementary Tables S3 and S4).

## Discussion

 In this study, we used data-driven ALFF and seed-based FC analysis to investigate the relationship between prolonged SL and aberrant intrinsic neural functional activity in ID patients who had sleep initiation problems. We identified that ID patients had higher ALFF value in the left insula and right amygdala and lower ALFF value in the right SPL. Compared to the HC, ID patients had significantly increased FC value in the left insula-precentral and right amygdala-left PCC. Pearson correlation analysis showed that the increased FC value between the right amygdala and left PCC was positively associated with prolonged SL measured by actigraphy, and there was a positive trend between increased ALFF values in the right amygdala and higher HAS scores. These correlations were still significant after adding the HAMA score as a covariate. The findings of this study revealed that disordered neurological activities in arousal and emotion-related brain regions were related to prolonged SL and arousal level in insomnia patients, which may be a potential neuroimaging mechanism of difficulty falling asleep.

 The insula is a key hub of the salience network (SN) and plays an important role in self-awareness, subjective salience, and cognitive processes [52]. Aberrant functional activity of the insula is considered an important physical marker of pathological anxiety and insomnia disorders, e.g., increased insula activity has been found to be correlated with anxiety and depression levels in insomniacs [53–55]. In line with the previous studies, we also found increased neural activity in the left insula, which may reflect the hyperarousal state of self-awareness and negative emotion processing in ID patients. However, the correlation analysis did not reveal a relationship between insula ALFF value and clinical characteristics.

 The precentral gyrus plays a major role in the sensorimotor network [56]. Huang et al. found increased FC between the precentral cortex and the amygdala in ID patients [37]. Goodman et al. indicated that the FC between the insula and precentral gyrus is fundamental to the processing of stressful experiences [57]. Furthermore, Killgore et al. showed increased FC between the precentral gyrus and primary sensory cortex in patients who have difficulty falling asleep [58]. In the current study, we similarly found increased FC between the left insula and bilateral precentral gyrus in ID patients. This may indicate a dysfunctional connection between emotional processing and sensorimotor brain regions in ID patients. However, no correlation was found between SL and FC of the insula and precentral gyrus in the current study.

 As an important part of the limbic system, the amygdala plays a critical role in initiating and processing adaptive behavioral responses to threats and arousal [59, 60]. Baglioni et al. reported that emotional arousal in ID patients is associated with enhanced amygdala activity [61]. Wassing et al. and Sun et al. found that decreased adaptation to negative stimuli in insomnia patients may be associated with amygdala dysregulation [62, 63]. In this study, we found a trend of positive correlations between increased activity in the right amygdala and higher HAS scores. The increased functional activity of the amygdala may induce heightened vigilance against external threats and inner impulses, which may cause a state of cognitive-psychological hyperarousal and then prolong SL time [64, 65]. In the current study, we found the ALFF value of the right amygdala was increased in ID patients compared with HCs, and the activation showed a positive correlation trend with arousal level. Thus, we suggest that increased right amygdala activity may be associated with abnormal emotion-cognitive control, which reveals an intrinsic mechanism of negative emotions in ID patients.

 The PCC, a core region of the DMN, has been found to be involved in self-referential processing, interoception and emotion regulation [60]. Zheng et al. found that patients with insomnia had significantly higher ALFF in PCC than HCs [18]. Yan et al. indicated that decreased activity in the left PCC was positively correlated with the insomnia severity index [66]. Furthermore, FC studies have proven that disruption of the right amygdala’s connectivity with the PCC could induce negative emotions [67, 68]. It was also demonstrated that emotional processing is overactive in insomnia patients, especially before falling asleep [69, 70]. Our results found that the FC between the right amygdala and left PCC was enhanced in ID patients, and the increased FC was positively related with SL measured by actigraphy. The correlation did not change after adjusting the anxiety level, which may imply the relationship between FC strength in the right amygdala-left PCC and SL time was not influenced by anxiety level in ID patients. Therefore, the enhanced FC between the amygdala and PCC might reflect a response to the “internal” threats and tense feelings in ID patients, and stronger connections between emotional and attention-processing brain regions may be an intrinsic neural mechanism for difficulty falling asleep.

 There are several limitations to our study. First, we did not find any functional alteration in the frontal areas. The reason may be attributed to clinical heterogeneity and the small size of the sample, especially for HCs, and future studies with larger sample sizes may be more representative. Second, no sleep poly-conductivity test was carried out in this study, so there was a lack of more accurate judgment on the quality and structure of sleep. Third, frequent awakenings during sleep and waking up early in the morning were not analyzed separately, and further studies in this area are necessary. Fourth, the present study is preliminary in exploring the neuroimaging mechanisms of difficulties falling asleep, and more in-depth mechanisms will be further explored in the future based on fMRI, polysomnography, and animal model experiments. Given these limitations, our research findings should be considered preliminary. ID subtype analysis and more accurate sleep monitoring should be used in future studies to further validate our findings.

## Conclusion

 This study represents a preliminary exploration of alterations in both regional fluctuations and inter-regional synchronization of spontaneous brain activity in ID patients with difficulty falling asleep. Our results demonstrated that ID patients exhibited higher ALFF values in the right amygdala, left insula, and right SPL. Increased FC strength was observed in the left insula-precentral and right amygdala-left PCC. Furthermore, the increased ALFF value of the right amygdala showed a positive correlation trend with the hyperarousal level, and the enhanced FC between the right amygdala and the left PCC was positively correlated with the SL measure by actigraphy. This study may shed light on the potential neural mechanisms underlying difficulty falling asleep in ID.

### Electronic supplementary material

Below is the link to the electronic supplementary material.

**Supplementary Material 1:** Correlation analysis results

## Data Availability

The raw data supporting the conclusions of this article can be obtained on reasonable request from the corresponding author. Requests to access the datasets should be directed to Jing Guo, guojing_2002@163.com.

## Abbreviations

ID: insomnia disorder
SL: sleep latency
FC: functional connectivity
fMRI: functional magnetic resonance imaging
HC: healthy controls
ALFF: Amplitude of low-frequency fluctuation
PSQI: Pittsburgh Sleep Quality Index
HAMA: Hamilton Anxiety Rating Scale
HAS: Hyperarousal Scale
SPL: superior parietal lobe
PCC: posterior cingulate cortex
DMN: default mode network
fALFF: fractional amplitude of low-frequency fluctuation
TCM: Traditional Chinese Medicine
DSM: Diagnostic and Statistical Manual of Mental Disorders
TR: repetition time
TE: echo time
FA: flip angle
FOV: field of view
EPI: echo planar imaging
CSF: cerebrospinal fluid
MNI: Montreal Neurological Institute
FD: framewise displacement
ROI: regions of interest
FDR: False Discovery Rate
L: Left
R: Right
