# Supplementary material for: Abnormal alterations of regional spontaneous neuronal activity and functional connectivity in insomnia patients with difficulty falling asleep: a resting-state fMRI study
Source: BMC Neurol. 2023 Dec 4;23:430. doi: 10.1186/s12883-023-03481-3 (PMC10694975; doi:10.1186/s12883-023-03481-3)
Supplement: Supplementary file 1 — Supplementary Material 1: Correlation analysis results [file 12883_2023_3481_MOESM1_ESM.docx]

**Supplementary.** **Correlation analysis results**

| **Table S1**. Correlation analysis between clinical scale test and ALFF in ID patients | | | | | | | | | | | | |
| --- | --- | --- | --- | --- | --- | --- | --- | --- | --- | --- | --- | --- |
|  | ALFF value in Insula _ L | | |  | ALFF value in Amygdala _ R | | | | ALFF value in SPL _ R | | | |
|  | r | *p_uncorrected_* | *p_Bonferroni-corrected_* |  | r | *p_uncorrected_* | | *p_Bonferroni-corrected_* |  | r | *p_uncorrected_* | *p_Bonferroni-corrected_* |
| PSQI | 0.053 | 0.708 | > 1.000 |  | -0.152 | | 0.282 | > 1.000 |  | -0.094 | 0.508 | > 1.000 |
| HAMA | -0.222 | 0.113 | 0.452 |  | 0.010 | | 0.944 | > 1.000 |  | -0.063 | 0.656 | > 1.000 |
| HAS | -0.059 | 0.680 | > 1.000 |  | 0.209 | | 0.026* | 0.104 |  | -0.034 | 0.808 | > 1.000 |
| SL | -0.174 | 0.216 | 0.864 |  | -0.145 | | 0.304 | > 1.000 |  | -0.036 | 0.802 | > 1.000 |

*: *p* < 0.05.

| **Table S2.** Correlation analysis between clinical scale test and FC in ID patients | | | | | | | | | | | | |
| --- | --- | --- | --- | --- | --- | --- | --- | --- | --- | --- | --- | --- |
|  | FC between  Insula_L and Precentral _ R | | |  | FC between  Insula_L and Precentral _ L | | | | FC between  Amygdala _ R and PCC _ L | | | |
|  | r | *p_uncorrected_* | *p_Bonferroni-corrected_* |  | r | *p_uncorrected_* | | *p_Bonferroni-corrected_* |  | r | *p_uncorrected_* | *p_Bonferroni-corrected_* |
| PSQI | -0.090 | 0.500 | > 1.000 |  | 0.064 | | 0.632 | > 1.000 |  | 0.249 | 0.076 | 0.304 |
| HAMA | -0.129 | 0.362 | > 1.000 |  | 0.0 | | 0.965 | > 1.000 |  | -0.045 | 0.750 | > 1.000 |
| HAS | 0.273 | 0.060 | 0.240 |  | 0.172 | | 0.192 | 0.768 |  | -0.074 | 0.603 | > 1.000 |
| SL | 0.051 | 0.699 | > 1.000 |  | -0.028 | | 0.833 | > 1.000 |  | 0.385 | 0.005* | 0.020* |

*: *p* < 0.05.

**Table S3.** Correlation analysis between clinical scale test and ALFF in ID patients after adding the HAMA score as covariates

|  | ALFF value in Insula _ L | | |  | ALFF value in Amygdala _ R | | | | ALFF value in SPL _ R | | | |
| --- | --- | --- | --- | --- | --- | --- | --- | --- | --- | --- | --- | --- |
|  | r | *p_uncorrected_* | *p_Bonferroni-corrected_* |  | r | *p_uncorrected_* | | *p_Bonferroni-corrected_* |  | r | *p_uncorrected_* | *p_Bonferroni-corrected_* |
| PSQI | 0.016 | 0.648 | > 1.000 |  | -0.124 | | 0.354 | > 1.000 |  | -0.134 | 0.315 | > 1.000 |
| HAS | -0.058 | 0.664 | > 1.000 |  | 0.306 | | 0.020* | 0.060 |  | -0.013 | 0.921 | > 1.000 |
| SL | -0.195 | 0.141 | 0.423 |  | -0.202 | | 0.128 | 0.384 |  | -0.010 | 0.942 | > 1.000 |

*: *p* < 0.05.

| **Table S4.** Correlation analysis between clinical scale test and FC in ID patients after adding the HAMA score as covariates | | | | | | | | | | | | |
| --- | --- | --- | --- | --- | --- | --- | --- | --- | --- | --- | --- | --- |
|  | FC between  Insula_L and Precentral _ R | | |  | FC between  Insula_L and Precentral _ L | | | | FC between  Amygdala _ R and PCC _ L | | | |
|  | r | *p_uncorrected_* | *p_Bonferroni-corrected_* |  | r | *p_uncorrected_* | | *p_Bonferroni-corrected_* |  | r | *p_uncorrected_* | *p_Bonferroni-corrected_* |
| PSQI | -0.059 | 0.622 | > 1.000 |  | 0.064 | | 0.633 | > 1.000 |  | 0.186 | 0.162 | 0.486 |
| HAS | 0.289 | 0.128 | 0.384 |  | 0.172 | | 0.196 | 0.588 |  | -0.085 | 0.526 | > 1.000 |
| SL | 0.076 | 0.572 | > 1.000 |  | -0.029 | | 0.828 | > 1.000 |  | 0.403 | 0.003* | 0.009* |

*: *p* < 0.05.
